# A double-blind comparative study of the safety and efficacy of caspofungin versus micafungin in the treatment of candidiasis and aspergillosis

**DOI:** 10.1007/s10096-012-1754-z

**Published:** 2012-10-03

**Authors:** S. Kohno, K. Izumikawa, M. Yoshida, Y. Takesue, S. Oka, K. Kamei, Y. Miyazaki, T. Yoshinari, N. A. Kartsonis, Y. Niki

**Affiliations:** 1 Department of Molecular Microbiology and Immunology, Nagasaki University Graduate School of Biomedical Sciences, 1-7-1 Sakamoto, Nagasaki, 852-8501 Japan; 2 Fourth Department of Internal Medicine, Teikyo University School of Medicine, Mizonokuchi Hospital, Kanagawa, Japan; 3 Department of Infection Control and Prevention, Hyogo College of Medicine, Hyogo, Japan; 4 AIDS Clinical Center, National Center for Global Health and Medicine, Tokyo, Japan; 5 Department of Clinical Research, Medical Mycology Research Center, Chiba University, Chiba, Japan; 6 Department of Chemotherapy and Mycoses, National Institute of Infectious Diseases, Tokyo, Japan; 7 Japan Development, Vaccine and Infectious Diseases, MSD K.K., Tokyo, Japan; 8 Clinical Research, Infectious Diseases, Merck Research Laboratories, Merck Research Institute, Upper Gwynedd, West Point, PA USA; 9 Development for Clinical Infectious Diseases, Showa University, Tokyo, Japan

## Abstract

The safety and efficacy profile of caspofungin and micafungin in Japanese patients with fungal infections were directly compared in this prospective, randomized, double-blind study. The proportion of patients who developed significant drug-related adverse event(s) (defined as a serious drug-related adverse event or a drug-related adverse event leading to study therapy discontinuation) was compared in 120 patients [caspofungin 50 mg, or 50 mg following a 70-mg loading dose on Day 1 (hereinafter, 70/50 mg) group: 60 patients; micafungin 150 mg: 60 patients]. The overall response rate was primarily evaluated in the per-protocol set (PPS) population. The proportion of patients who developed significant drug-related adverse events was 5.0 % (3/60) in the caspofungin group and 10.0 % (6/60) in the micafungin group [95 % confidence interval (CI) for the difference: −15.9 %, 5.2 %]. The favorable overall response in the PPS population for patients with esophageal candidiasis, invasive candidiasis, and chronic pulmonary aspergillosis including aspergilloma was 100.0 % (6/6), 100.0 % (3/3), and 46.7 % (14/30) in the caspofungin group, and 83.3 % (5/6), 100.0 % (1/1), and 42.4 % (14/33) in the micafungin group, respectively. In Japanese patients with *Candida* or *Aspergillus* infections, there was no statistical difference in the safety between caspofungin and micafungin. Consistent with other data on these two agents, the efficacy of caspofungin and micafungin was similar.

## Introduction

The importance of deep-seated fungal infections in Japan is considered to be increasing due to the rise in the number of immunocompromised patients associated with the introduction of advanced medical treatment and the aging of the Japanese population as a whole. *Candida* spp. and *Aspergillus* spp. are the most important causative pathogens in Japan, the same as in other countries [1, 2].

Echinocandins inhibit the biosynthesis of (1,3)-β-D-glucan, the structural component of fungal cell wall, thereby, exhibiting antifungal activity against *Candida* spp. and *Aspergillus* spp. Although caspofungin, micafungin sodium (hereinafter, micafungin), and anidulafungin have been approved and are used worldwide, micafungin is the only approved echinocandin antifungal agent in Japan at the time of this study.

Caspofungin has been shown to be effective as the primary therapy for esophageal candidiasis and invasive candidiasis, as salvage therapy for invasive aspergillosis, and as empirical therapy in patients with persistent fever and neutropenia. To date, caspofungin has been approved for use in over 80 countries worldwide, including the United States and Europe [3–6]. A comparator-controlled study of caspofungin and micafungin conducted in patients with candidemia has been reported by Pappas et al. In this study, micafungin 100 mg or 150 mg once daily was shown to be effective (non-inferior) compared to caspofungin 50 mg daily following a 70-mg loading dose on Day 1 [7]. Additionally, in a cohort analysis, caspofungin and micafungin were compared as empirical therapy in patients with febrile neutropenia, with similar efficacy reported [8]. There are no reports on the comparative study of caspofungin and micafungin for aspergillosis.

Herein, we report the results of a randomized, double-blinded, comparative study of caspofungin versus micafungin conducted in Japanese patients with *Candida* or *Aspergillus* infections. The safety and efficacy profiles of caspofungin and micafungin were compared.

## Study patients and study plan

### Objective and study design

This is a randomized, multicenter, double-blind, comparative study. The study was conducted in 43 study sites in Japan from August 2008 through July 2010. The protocol was reviewed by the Institutional Review Board of each participating site, and written informed consent was obtained from each patient. The protocol was also registered on clinicaltrials.gov (NCT00717860). In this study, a serious drug-related adverse event or a drug-related adverse event leading to study therapy discontinuation was defined as significant drug-related adverse event(s). Definitions of adverse events and drug relationships, and the determination of seriousness basically complied with the “Definitions and Terminology Associated with Clinical Safety Experience” in the International Conference on Harmonisation (ICH)-E2 [9]. The primary objective of this study was to compare the difference in the proportion of patients who develop significant drug-related adverse events(s) between the caspofungin and micafungin groups. The secondary objective was to evaluate the difference in the overall response by each of esophageal candidiasis, invasive candidiasis, and aspergillosis.

### Patient inclusion criteria

Japanese patients aged 20 years and over were enrolled in this study following obtainment of written informed consent. Patients who fulfilled the criteria indicated below were enrolled as probable disease cases. When causative fungi (*Candida* spp. or *Aspergillus* spp.) were identified by culture or relevant organisms with specific morphology (yeast or acutely branching mold with septated hyphae) were observed by microscopic examination in addition to the criteria below, then patients were enrolled as proven disease cases. Both probable and proven disease cases were the target population in this study.

Criteria for probable disease:

- Esophageal candidiasis: patients with clinical symptoms of esophageal candidiasis (i.e., odynophagia, dysphagia, and heartburn) and plaque observed on the esophageal mucosa by endoscopy.
- Candidemia: patients with fever >38 °C observed, or fever of ≥37.5 °C that continues for 1 h or more despite the use of antibiotic therapy and positive results for the (1,3)-β-D-glucan test.
- Other types of invasive candidiasis (except candidemia): fungal infection strongly suspected at screening based on the clinical course and symptoms, typical radiographic imaging findings on X-ray and computed tomography (CT) (based on infection site), and positive results for the (1,3)-β-D-glucan test.
- Invasive aspergillosis: patients with risk factors of fungal infections (e.g., neutropenia, immunosuppressive treatment), clinical symptoms (e.g., fever, generalized malaise, coughing, sputum, bloody sputum, dyspnea), characteristic radiographic imaging findings (e.g., infiltration shadow, nodular shadow, cavitary lesions, or halo sign), and positive results for *Aspergillus* galactomannan antigen (enzyme-linked immunosorbent assay).
- Chronic pulmonary aspergillosis (except pulmonary aspergilloma): patients with clinical symptoms (e.g., fever not responding to antibiotic agent, body weight decreased, wet coughing, bloody sputum), characteristic radiographic imaging findings (e.g., pericavity infiltration, increasing size of cavity, or fluid collection in the cavity), and positive results for *Aspergillus* antibody or *Aspergillus* galactomannan antigen.
- Pulmonary aspergilloma: patients who have clinical symptoms (e.g., sputum, bloody sputum, hemoptysis, fever, dyspnea, coughing), characteristic radiographic imaging findings (e.g., coccus image in the cavity, thickened cavity wall, pleural thickening, or fluid collection in the cavity), and positive results for *Aspergillus* antibody.

Of note, patients who received prior antifungal therapies (other than echinocandins) were also allowed to enroll in this study. In such cases, the patients were evaluated on whether they met the criteria of refractoriness (the patient received an antifungal agent within 7 days prior to study therapy administration, but the disease progressed or clinical improvement was not observed) or intolerance (there is a significant problem in tolerance during the administration of prior antifungal agents as judged by the investigators).

Patients who fall under any of the criteria listed below were to be excluded: patients with mycoses due to causes other than *Candida* spp. and *Aspergillus* spp.; patients who had already received caspofungin or micafungin for the current fungal infection within the 7 days prior to initiation of the study; International Normalized Ratio (INR) (prothrombin time) of >2 × ULN (upper limit of normal) for patients not receiving anticoagulants; INR >4 × ULN for patients receiving anticoagulants; total bilirubin of >5 × ULN; aspartate aminotransferase (AST), alanine aminotransferase (ALT), or alkaline phosphatase (ALP) of >5 × ULN; patients with a history of serious drug-related allergy or sensitivity; patients with moderate or severe hepatic insufficiency (acute hepatitis, hepatic cirrhosis, etc.); patients who received another investigational drug within 1 month prior to study entry; patients who are pregnant, intend to become pregnant during the period up to 2 weeks after study completion, or are lactating.

### Treatment plan

The randomization was stratified by infection category [esophageal candidiasis, candidemia, other types of invasive candidiasis (except candidemia), invasive aspergillosis, chronic pulmonary aspergillosis, and pulmonary aspergilloma] using a random permuted block, with the caspofungin group and micafungin group allocated at a ratio of 1:1. Patients, study investigators, and the sponsor remained blinded to the treatment group throughout the study. The pharmacist or preparer of the study therapy at each site was not blinded to the treatment group, but this individual could not be involved with any evaluation or judgment of efficacy and safety in this study.

Each patient received intravenous administration of caspofungin (esophageal candidiasis: 50 mg, invasive candidiasis and aspergillosis: 70/50 mg) once daily or micafungin 150 mg once daily for approximately 1 h in a blinded fashion. The treatment periods were 7–28 days for patients with esophageal candidiasis, 14–56 days for patients with invasive candidiasis, and 14–84 days for patients with aspergillosis. Patients with esophageal candidiasis were treated with study therapy for at least 3 days after the resolution of clinical symptoms and signs. Patients with candidemia were treated for at least 14 days after the last positive culture result for *Candida* spp. Patients with aspergillosis were treated for at least 7 days after the resolution of clinical symptoms/signs and at least 14 days after the resolution of neutropenia (absolute neutrophil count; ANC: >500/μL). The use of other systemic antifungal agents and rifampin was prohibited until the time of the efficacy evaluation.

### Safety and efficacy evaluation

With regard to the safety of the study drug, the investigators recorded all adverse events and drug-related adverse events occurring from the initiation of study therapy through 14 days after the last dose of the study drug, based on any abnormal physical findings, vital signs, and laboratory tests, including red blood cell count, white blood cell count, hemoglobin, hematocrit, platelet count, total protein, albumin, total bilirubin, direct bilirubin, AST, ALT, γ-glutamyl transpeptidase (γ-GTP), ALP, lactate dehydrogenase, blood urea nitrogen, creatinine, Na, K, Cl, Ca, uric acid, blood glucose, C-reactive protein, urinalysis, prothrombin time, and partial thromboplastin time. All safety information pertaining to a significant drug-related adverse event was reviewed by the Independent Safety Assessment Committee (ISAC) under blinded conditions for study therapy. In addition, with regard to hepatic function tests, maximum values from the study period were graded according to the Common Terminology Criteria for Adverse Events (CTCAE) Version 3 [10].

The diagnosis of patients enrolled into this study was reviewed by an Independent Efficacy Assessment Committee (IEAC). The efficacy results in this study were based on the overall response, which included the resolution or improvement of clinical symptoms and radiographic imaging findings [or eradication of *Candida* (microbiological response) in patients with candidemia]). All efficacy evaluations made by the investigators were reviewed by the IEAC in a blinded fashion, and the judgment by the IEAC was considered as the final result.

The efficacy evaluation in esophageal candidiasis was conducted 5–7 days after the end of study therapy. The overall response was determined as “favorable” in patients with esophageal candidiasis if clinical symptoms and signs of *Candida* infections (odynophagia, dysphagia, and heartburn) resolved and follow-up endoscopy results indicated at least a two-grade improvement (or return to Grade 0) in the predefined criteria (Grades 0, 1/2, 1, 2, 3, and 4) [11]. The efficacy evaluation in invasive candidiasis was conducted at the completion of study therapy. The overall response was determined to be “favorable” in patients with invasive candidiasis if the clinical symptoms and signs of *Candida* infections were resolved and follow-up blood culture was negative (for patients with candidemia) or follow-up radiographic imaging findings were “improved” [for patients with other types of invasive candidiasis (except candidemia)]. The efficacy evaluation in aspergillosis was conducted at the completion of study drug. The overall response was determined to be “favorable” in patients with aspergillosis if the clinical symptoms and signs of *Aspergillus* infections were “improved” or “stable”, and follow-up radiographic imaging findings were “improved” or “stable”. However, if the clinical symptoms and signs and radiographic imaging findings were both “stable” in patients with aspergillosis, the overall response was determined to be “unfavorable”.

### Identification of fungus and drug sensitivity study

Fungus isolated in the study was sent to Mitsubishi Chemical Medience Corporation and the organism was identified to the species level. The susceptibility of all isolated *Aspergillus* spp. and *Candida* spp. to antifungal agents was measured according to the guidance for microdilution technique M38-A2 (*Aspergillus* spp.) [12] and M27-A3 (*Candida* spp.) [13] of the Clinical and Laboratory Standards Institute (CLSI).

### Statistical analysis

The safety analysis population was the all patients as treated (APaT) population (all randomized patients who received at least one dose of study therapy). The incidence and its 95 % confidence interval (CI) by treatment groups were calculated for the primary endpoint, namely, the proportion of patients who developed significant drug-related adverse events (a serious drug-related adverse event or a drug-related adverse event leading to study therapy discontinuation). In addition, 95 % CIs for the difference in the incidence between treatment periods were calculated using the Miettinen and Nurminen method (1985). The study was not powered to show a statistically significant difference between treatment groups.

The primary efficacy analysis population was the per-protocol set (PPS) population. The PPS included any patient who was diagnosed as having *Candida* or *Aspergillus* infections by the IEAC and received an appropriate course of study therapy (at least 5 days for the treatment of esophageal candidiasis or invasive candidiasis or at least 7 days for the treatment of aspergillosis), and in whom the efficacy evaluation was conducted in accordance with the study protocol. For esophageal candidiasis and candidemia, patients were included in the PPS population only when *Candida* spp. was confirmed by culture test. In addition, a secondary efficacy analysis was also performed using the full analysis set (FAS) population to confirm the consistency of the results. The FAS included any patient who received at least one dose of study therapy and was diagnosed as having *Candida* or *Aspergillus* infections by the IEAC.

Patients whose overall response was determined as “unable to be judged” were excluded from the overall response in the PPS analysis. In the FAS analysis, “unable to be judged” patients were treated as “unfavorable”. The proportion of patients with a favorable overall response and its 95 % CI were calculated by three disease types (esophageal candidiasis, invasive candidiasis, and chronic pulmonary aspergillosis including aspergilloma), as judged by the IEAC. The analysis methods, handling, and the identification of the patients to be excluded from the PPS population mentioned above were determined before the unblinding.

## Results

### Study patients and patient background

One hundred and twenty-one patients were randomized. The average age of the randomized patients at the time of enrollment was 69.1 years and the proportion of male patients (79.3 %) was greater than that of female patients (20.7 %). The average weight was 48.8 kg and patients who were refractory to or intolerant of prior antifungal agents accounted for approximately one-quarter of enrollment. There were no patients with human immunodeficiency virus (HIV) infection, allogeneic stem cell transplant, or graft versus host disease. Major risk factors observed in patients with esophageal candidiasis were diabetes mellitus (25.0 %) and malignant tumor (25.0 %). Major risk factors in patients with invasive candidiasis were diabetes mellitus (31.6 %) and malignant tumor (26.3 %). Major risk factors in patients with chronic pulmonary aspergillosis were pulmonary disorder (31.4 %), tuberculosis sequelae (24.3 %), diabetes mellitus (21.4 %), malignant tumor (8.6 %), and use of steroids (5.7 %). There was no statistical difference between the caspofungin group and the micafungin group for any demographic or baseline data (Table 1)

**Table 1:** Patient demographics and background conditions (all randomized patients)

	Total		Caspofungin		Micafungin		*p*-value^a^
	*n*	(%)	*n*	(%)	*n*	(%)	
Randomized patients	121		61		60		
Sex							0.472
Male	96	(79.3)	50	(82.0)	46	(76.7)	
Female	25	(20.7)	11	(18.0)	14	(23.3)	
Age (years)							0.815
Mean	69.1		68.9		69.3		
Standard deviation	10.1		11.2		9.0		
Weight (kg)							0.476
Mean	48.80		49.56		48.01		
Standard deviation	11.61		10.75		12.47		
Refractoriness or intolerance to prior antifungal agents							0.884
Refractory	23	(19.0)	12	(19.7)	11	(18.3)	
Intolerant	5	(4.1)	2	(3.3)	3	(5.0)	
Primary therapy	93	(76.9)	47	(77.0)	46	(76.7)	
Underlying risks							0.478^c^
Diabetes mellitus	28	(23.1)	11	(18.0)	17	(28.3)	
Pulmonary disorder^b^	25	(20.7)	13	(21.3)	12	(20.0)	
Malignant tumor	22	(18.2)	13	(21.3)	9	(15.0)	
Tuberculosis sequelae	20	(16.5)	9	(14.8)	11	(18.3)	
Use of immunosuppressive drugs	5	(4.1)	1	(1.6)	4	(6.7)	
Use of steroids	5	(4.1)	2	(3.3)	3	(5.0)	
Neutrophil count <500/mm^3^	4	(3.3)	2	(3.3)	2	(3.3)	
Thermal burn	1	(0.8)	1	(1.6)	0	(0.0)	

^a^Chi-square test (*t*-test for age and weight)

^b^Pulmonary disorder includes bronchiectasis, tuberculosis, chronic obstructive pulmonary disease, pulmonary fibrosis, and pulmonary bulla

^c^Based on the comparison of the proportion of patients who have at least one of the underlying risks between two treatment groups

**Fig. 1:**
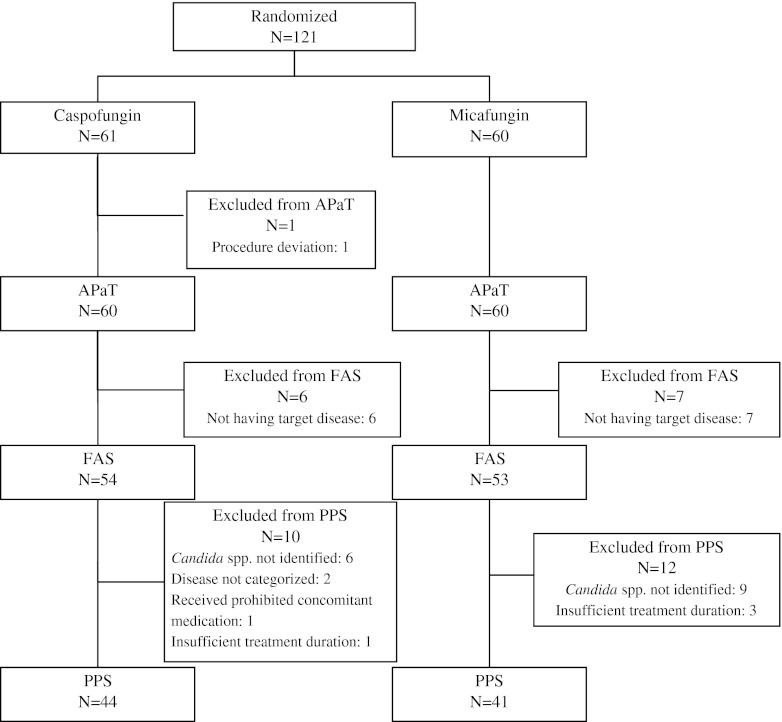
Analysis populations and reasons for exclusion by treatment group. APaT: all patients as treated, FAS: full analysis set, PPS: per-protocol set

The breakdown of APaT, FAS, PPS and populations in this study and the reasons for the exclusion of patients from each population is included in Fig. 1. One patient was excluded from the APaT population because blinding was not maintained for this patient. Thirteen patients who were diagnosed as having infections caused by pathogens other than *Aspergillus* spp. and *Candida* spp., based on the determination of the IEAC, were excluded from the FAS population. The most common reason for why patients were excluded from the FAS population and the PPS population was unconfirmed “positive culture” for esophageal candidiasis and invasive candidiasis (15 patients). Most of these excluded patients were with probable candidemia. Candidemia patients were allowed to start study therapy based on the positive (1,3)-β-D-glucan test and clinical symptoms, and, as a result, most of the culture results in these patients were demonstrated as negative. Patients who were not classified into diseases predefined in the study protocol (two patients with aspergillosis not classified) were also excluded from the PPS population. In addition, there were exclusions due to the use of prohibited concomitant drugs (one patient) and insufficient study therapy duration (four patients). There was no notable difference in the number of patients within each treatment group in any of the three analysis populations.

The average dosages in the APaT population were 51.0 mg/day and 149.7 mg/day in the caspofungin and micafungin groups, respectively. The average duration of study drug treatment in the APaT population was 28.7 (range 2–84) days and 33.6 (range 1–84) days in the caspofungin and micafungin groups, respectively. The accounting of patients by disease type is presented in Table 2.

**Table 2:** Disposition of patients by disease type

Disease type^a^	APaT		PPS	
	Number of patients [duration of therapy, mean days]		Number of patients	
	Caspofungin	Micafungin	Caspofungin	Micafungin
Esophageal candidiasis	9 [14.7]	7 [13.7]	8	6
Invasive candidiasis	9 [13.2]	9 [13.2]	3	1
Candidemia	6	7	1	0
Invasive candidiasis (excluding candidemia)	3	2	2	1
Aspergillosis	36 [37.1]	37 [42.1]	33	34
Invasive aspergillosis	1	0	1	0
Chronic pulmonary aspergillosis (including pulmonary aspergilloma)	33	37	32	34
Pulmonary aspergillosis (unclassified)	2	0	0	0
Other than mycosis^b^	6 [21.8]	7 [34.9]	0	0
Total	60 [28.7]	60 [33.6]	44	41

*APaT* all patients as treated, *PPS* per-protocol set

^a^Disease classification is based on the diagnosis by the Independent Efficacy Assessment Committee (IEAC)

^b^Other infectious diseases (not mycosis) diagnosed by the IEAC

### Safety evaluation

The number of patients who reported drug-related adverse events is shown in the APaT population in Table 3. Drug-related adverse events were reported in 38.3 % and 41.7 % of patients in the caspofungin and micafungin groups, respectively. Serious drug-related adverse events were reported in two patients; both were in the micafungin group (AST and ALT increased in one patient and rash in the other patient).

**Table 3:** The number (%) of patients with clinical and laboratory drug-related adverse events (incidence ≥3 % in one or more treatment groups) [all patients as treated (APaT) population]

	Caspofungin^a^		Micafungin^b^	
	*n*	(%)	*n*	(%)
Patients in population	60		60	
With one or more drug-related adverse events	23	(38.3)	25	(41.7)
With one or more drug-related serious adverse events	0	(0.0)	2	(3.3)
Eye disorders	1	(1.7)	2	(3.3)
Gastrointestinal disorders	3	(5.0)	4	(6.7)
Constipation	0	(0.0)	2	(3.3)
Nausea	2	(3.3)	1	(1.7)
General disorders and administration site conditions	2	(3.3)	3	(5.0)
Injection site reaction	0	(0.0)	2	(3.3)
Hepatobiliary disorders	1	(1.7)	2	(3.3)
Infections and infestations	0	(0.0)	2	(3.3)
Laboratory abnormalities	14	(23.3)	18	(30.0)
Alanine aminotransferase (ALT) increased	5	(8.3)	4	(6.7)
Aspartate aminotransferase (AST) increased	6	(10.0)	3	(5.0)
Blood lactate dehydrogenase (LDH) increased	0	(0.0)	2	(3.3)
Blood potassium decreased	2	(3.3)	1	(1.7)
Blood potassium increased	1	(1.7)	3	(5.0)
Blood pressure increased	0	(0.0)	2	(3.3)
Eosinophil count increased	3	(5.0)	4	(6.7)
Gamma-glutamyl transpeptidase (γ-GTP) increased	2	(3.3)	2	(3.3)
Prothrombin time prolonged	2	(3.3)	0	(0.0)
White blood cell count decreased	1	(1.7)	2	(3.3)
White blood cell count increased	0	(0.0)	2	(3.3)
Platelet count increased	0	(0.0)	2	(3.3)
Blood alkaline phosphatase (ALP) increased	2	(3.3)	2	(3.3)
Nervous system disorders	3	(5.0)	2	(3.3)
Hypoesthesia	0	(0.0)	2	(3.3)
Skin and subcutaneous tissue disorders	1	(1.7)	6	(10.0)
Erythema	0	(0.0)	2	(3.3)
Rash	1	(1.7)	3	(5.0)
Vascular disorders	5	(8.3)	2	(3.3)
Hypertension	2	(3.3)	0	(0.0)
Phlebitis	2	(3.3)	2	(3.3)

^a^Patients with esophageal candidiasis received caspofungin 50 mg once daily. All other patients received caspofungin 50 mg once daily following a 70-mg loading dose on Day 1

^b^All patients received micafungin 150 mg once daily

Every patient is counted once for each applicable specific adverse event. A patient with multiple adverse events within a system organ class is counted once for that system organ class. A system organ class or specific adverse event appears in this table only if its incidence in one or more of the columns is greater than or equal to the percent incidence specified in the report title, after rounding

Abnormal values in ALT, AST, and ALP (maximal levels), regardless of the drug relationship, were assessed in an exploratory fashion in accordance with CTCAE Version 3. The numbers of patients who had Grade 2 or higher ALT, AST, or ALP elevations (>2.5 × ULN) were 3, 4, and 5, respectively, in the caspofungin group, and 6, 5, and 2, respectively, in the micafungin group. Of these, the number of patients who had Grade 3 ALT, AST, or ALP elevations (>5.0–20.0 × ULN) was 2, 3, and 1, respectively, in the micafungin group; none of the caspofungin-treated patients had Grade 3 elevations for ALT, AST, or ALP.

The proportion of patients fulfilling the primary endpoint of this study, the presence of one or more significant drug-related adverse events, was 5.0 % (95 % CI: 1.0, 13.9) in the caspofungin group and 10.0 % (95 % CI: 3.8, 20.5) in the micafungin group. The between-treatment difference was −5.0 % (95 % CI: −15.9, 5.2), thereby, showing no significant difference between the two groups. Significant drug-related adverse events were reported in three patients in the caspofungin group (all reported drug-related adverse events leading to study therapy discontinuation) and six patients in the micafungin group (two reported serious drug-related adverse events accompanied by study therapy discontinuation and four reported drug-related adverse events leading to study therapy discontinuation). The significant adverse events of three patients in the caspofungin group were elevation of ALP, AST, and γ-GTP, moderate rash, and elevation of AST and ALT. The significant adverse events in six patients of the micafungin group were elevation of AST and ALT, moderate rash, increased blood pressure level, occurrence of atrial fibrillation, elevation of γ-GTP alone, and elevation of AST, ALT, γ-GTP, ALP, and LDH with the occurrence of nausea. Nine patients in the caspofungin group and 10 patients in the micafungin group died during this study. None of the deaths were considered to be drug-related adverse events.

### Efficacy evaluation

Of the 85 patients included in the PPS population, six patients were deemed to be “unable to be judged”, and the favorable overall response rate was assessed for 79 patients. Favorable overall response rates in esophageal candidiasis, invasive candidiasis, and chronic pulmonary aspergillosis including aspergilloma are shown in Table 4. Among invasive candidiasis, one patient in the caspofungin group was candidemia and the others (two in caspofungin and one in micafungin) were peritoneal candidiasis patients. The overall response of caspofungin and micafungin in chronic pulmonary aspergillosis (other than aspergilloma) patients were 45.0 % (9/20) and 46.7 % (14/30), respectively. The overall response of caspofungin in aspergilloma patients was 50.0 % (5/10), and there were no aspergilloma patients in the micafungin group. In general, the favorable overall responses were similar across the two treatment groups for each disease. Since the efficacy evaluation was independently assessed from an event of death, a listing of patients in the PPS population who died during the study period is shown in Table 5. Three of the four patients in the caspofungin group and one of four patients in the micafungin group died due to the worsening of primary infection (chronic pulmonary aspergillosis in all cases). Three patients deemed to be “unable to be judged” were not included in the calculation of the favorable overall response rate (two patients in the caspofungin group and one patient in the micafungin group).

**Table 4:** Overall response in the per-protocol set (PPS) excluding patients deemed to be “unable to be judged”^a^ from the PPS population

	Caspofungin		Micafungin	
Number of patients in PPS	44		41	
Number of patients determined as “unable to be judged” for overall response	5		1	
Number of patients analyzed for overall response	39		40	
Overall response	Favorable response rate, % (n/m)^b^	(95 % CI)	Favorable response rate, % (n/m)^b^	(95 % CI)
Esophageal candidiasis	100.0 (6/6)	(54.1, 100.0)	83.3 (5/6)	(35.9, 99.6)
Invasive candidiasis	100.0 (3/3)	(29.2, 100.0)	100.0 (1/1)	(2.5, 100.0)
Chronic pulmonary aspergillosis including aspergilloma	46.7 (14/30)	(28.3, 65.7)	42.4 (14/33)	(25.5, 60.8)

*CI* confidence interval

^a^Patients who were determined as “unable to be judged” were excluded from the PPS analysis for overall response

^b^ *n*/*m* number of patients with favorable overall response/number of patients analyzed

**Table 5:** Listing of patients who died in the PPS population

Treatment group	Disease	Study therapy duration	Overall response (by the IEAC)	Date of death (relative day after study therapy completion)	Cause of death (by primary investigators)
Caspofungin	Chronic pulmonary aspergillosis	11 days	Unable to judge (due to severe co-infection of bacteria)	Day 1	(Worsening of) chronic pulmonary aspergillosis
	Chronic pulmonary aspergillosis	84 days	Unfavorable	Day 11	(Worsening of) chronic pulmonary aspergillosis
	Chronic pulmonary aspergillosis	84 days	Unable to judge (due to repeated co-infection of bacteria)	Day 12	(Worsening of) chronic pulmonary aspergillosis
	Candidemia	15 days	Favorable	Day 11	(Worsening of) peritoneal mesothelioma
Micafungin	Chronic pulmonary aspergillosis	8 days	Unable to judge (due to inconsistent imaging data)	Day 2	(Worsening of) lung cancer
	Chronic pulmonary aspergillosis	20 days	Unfavorable	Day 7	(Worsening of) chronic pulmonary aspergillosis
	Chronic pulmonary aspergillosis	8 days	Unfavorable	Day 19	(Worsening of) COPD
	Chronic pulmonary aspergillosis	13 days	Unfavorable	Day 8	Death (unknown cause of death)

Additionally, in the FAS population, the favorable overall response rates in the caspofungin group and the micafungin group were 77.8 % (7/9) and 85.7 % (6/7) for patients with esophageal candidiasis, 33.3 % (3/9) and 11.1 % (1/9) for patients with invasive candidiasis, 45.5 % (15/33) and 37.8 % (14/37) for patients with chronic pulmonary aspergillosis including aspergilloma, respectively. The results were generally comparable between the treatment groups, such as those seen in the PPS population.

### Duration of therapy and relationships with overall response in aspergillosis patients

Among patients with aspergillosis in the PPS population, an exploratory assessment was performed to compare the number of days on study therapy between the treatment groups and by treatment outcome. The mean number (range) of days on study therapy among patients with a favorable response was 36.1 (8 to 84) days for the caspofungin 70/50 mg group (*n* = 14) and 61.5 (22 to 84) days for the micafungin group (*n* = 14). The mean treatment duration was shorter among patients with a favorable response in the caspofungin 70/50 mg group than in the micafungin group. On the other hand, the mean number (range) of days on study therapy among patients with an unfavorable response with aspergillosis was 39.3 (14 to 84) days for the caspofungin 70/50 mg group (*n* = 16) and 35.6 (7 to 84) days for the micafungin group (*n* = 19). The treatment duration was generally comparable between the groups in patients with unfavorable responses.

### Susceptibility of fungal isolates to caspofungin

The geometric mean (range) of the caspofungin minimum inhibitory concentration (MIC) of clinical isolates of *Candida* spp. detected at screening (baseline isolates) was 0.25 (0.06–0.5) μg/mL and 0.5 μg/mL for *C. albicans* (19 strains) and *C. glabrata* (one strain), respectively. The geometric mean (range) of the caspofungin minimum effective concentration (MEC) of clinical isolates of *Aspergillus* spp. detected at screening was 0.25 (0.12–0.5) μg/mL, 0.25 (0.25) μg/mL, 0.25 (0.12–0.5) μg/mL, and 0.12 μg/mL for *A. fumigatus* (nine strains), *A. niger* (three strains), *A. flavus* (two strains), and *Aspergillus* spp. (one strain), respectively.

## Discussion

This study is a prospective, randomized, double-blind study to evaluate the efficacy and safety of caspofungin versus micafungin in Japanese patients with *Aspergillus* or *Candida* infections. The caspofungin doses investigated in this study were the same as the approved clinical doses outside of Japan. Although the approved standard dose of micafungin for aspergillosis and candidiasis is 50–150 mg once daily and 50 mg once daily, respectively, and the dose can be increased up to 300 mg once daily in Japan, the average daily micafungin dose which has been actually used in a clinical setting is reported to be 110 mg [14]. In addition, in the Japanese “Diagnosis and Treatment Guideline for Deep-Seated Fungal Infections”, micafungin doses of 100 to 150 mg daily and 150 to 300 mg daily are recommended for the treatment of candidiasis and aspergillosis, respectively [15]. Based on these data, a micafungin dose of 150 mg daily was determined to be an appropriate comparison to caspofungin (50 mg or 70/50 mg once daily).

Several efficacy findings deserve further attention. The efficacy results from the patients who were in the PPS excluding “unable to be judged” patients (*n *= 79) suggest that the efficacy of caspofungin 50 mg or 70/50 mg once daily was almost comparable to that of micafungin 150 mg once daily. However, it should be noted that two patients in the PPS population receiving caspofungin died due to worsening of the primary disease of chronic pulmonary aspergillosis after 1 and 12 days following the completion of study therapy, respectively, and were assessed as “unable to be judged” by the IEAC because both patients also had bacterial infection and the efficacy of caspofungin could not be evaluated based on their clinical symptoms. Since the ultimate cause of death was the worsening of primary disease, these two patients were highly likely not to respond to caspofungin, and, consequently, the efficacy of caspofungin might be slightly lower in this study. All *Candida* spp. isolates detected at screening in this study showed caspofungin MIC below the current CLSI clinical breakpoint (2 μg/mL) and were deemed to be susceptible. No CLSI clinical breakpoint for *Aspergillus* spp. has been established; however, the MEC values were similar to the data reported to date [16]. Therefore, *Candida* spp. and *Aspergillus* spp. in Japan appear to be susceptible to caspofungin.

Both caspofungin and micafungin demonstrated favorable treatment efficacy against *Candida* infections. This result is similar to that in the fluconazole-controlled comparative studies of caspofungin and micafungin in patients with esophageal candidiasis [3, 17] and to that in the direct comparative study between caspofungin and micafungin in patients with invasive candidiasis [7].

On the other hand, the favorable response rate was slightly below 50 % in aspergillosis. In this study, no patients in the primary efficacy analysis group were confirmed by the IEAC to have invasive aspergillosis, and, thus, all patients who were categorized into the aspergillosis population had subacute to chronic stage of aspergillosis. As for the study evaluating the efficacy against chronic pulmonary aspergillosis, a study has been conducted comparing micafungin with voriconazole. In this study, the favorable overall response rates at the completion of study therapy with micafungin (average dose 167.4 mg/day) or voriconazole (6 mg/kg twice daily on Day 1, followed by 4 mg/kg twice daily on Day 2 onwards) were 60.0 % and 53.2 %, respectively [18]. Although a direct comparative assessment is difficult due to the different enrollment and efficacy evaluation criteria, based on this previous report and the results from the current study (favorable overall response rate of 46.7 % in the caspofungin group and 42.4 % in the micafungin group), it can be considered that both agents are effective to some extent against chronic pulmonary aspergillosis. Additionally, among the chronic pulmonary aspergillosis patients who showed favorable efficacy response, we found that the duration of therapy in the caspofungin group was numerically shorter than that in the micafungin group. Since the number of patients was very limited (*n* = 14 in each group) and any adjustment based on the medical history or concomitant diseases including risk factors for fungal infection was not considered, it is difficult to conclude that the difference in periods show antifungal responses. However, it might be interesting to investigate the difference of echinocandins, and, thus, further investigation in the patients with more controlled status is needed.

Taken together, the overall efficacy results seem consistent to those of other previous reports, although there is a limitation to comparing the efficacy to each candidiasis and aspergillosis between caspofungin and micafungin due to the small number of patients in each subset of infection.

Amongst the proportion of patients with significant drug-related adverse events, the primary endpoint of this study was 5.0 % in the caspofungin group and 10.0 % in the micafungin group. The 95 % CI for the treatment difference in the incidence was −15.9 % to 5.2 %, thereby, showing no significant difference. Furthermore, no apparent difference between the treatment groups was observed in the incidence of specific adverse events or drug-related adverse events. In addition, relatively common drug-related adverse event categories were similar to those previously reported in association with caspofungin [3–5]. Drug-related adverse events relating to liver function enzymes have been commonly reported in association with echinocandins. Since these events were also frequently reported compared to other drug-related adverse events in this study, these events were further assessed. When maximal levels of AST, ALT, and ALP were graded in accordance with CTCAE Version 3 criteria, all abnormal changes observed in the caspofungin group were Grade 2 (>2.5–5 × ULN), but some patients in the micafungin group had Grade 3 levels (>5.0–20.0 × ULN). Since multiple types of drugs were concomitantly used with the study therapy in this trial, a discussion of the drug association with elevation of these enzymes is difficult to make. However, the monitoring of liver function enzymes is generally recommended for patients receiving echinocandins.

## Conclusion

In Japanese patients with *Aspergillus* or *Candida* infections, the safety of the treatment with caspofungin 50 mg or 70/50 mg once daily was similar to that of micafungin 150 mg daily. Consistent to other data on these two agents, caspofungin treatment showed similar efficacy to micafungin.
